# Length of prostate biopsies is not necessarily compromised by pooling multiple cores in one paraffin block: an observational study

**DOI:** 10.1186/s12907-015-0001-9

**Published:** 2015-03-08

**Authors:** Teemu T Tolonen, Jorma Isola, Antti Kaipia, Jarno Riikonen, Laura Koivusalo, Sanna Huovinen, Marita Laurila, Sinikka Porre, Mika Tirkkonen, Paula Kujala

**Affiliations:** Department of Pathology, Fimlab Laboratories, Tampere University Hospital, Tampere, Finland; Department of Cancer Biology, Institute of Biomedical Technology, University of Tampere, Tampere, Finland; Department of Surgery, Satakunta Hospital district, Pori, Finland; Department of Urology, Tampere University Hospital, Tampere, Finland; Department of Materials Science, Tampere University of Technology, Tampere, Finland

**Keywords:** Prostate cancer, Prostatic needle biopsies, Biopsy quality, Guidelines

## Abstract

**Background:**

Individually submitted prostatic needle biopsies are recommended by most guidelines because of their potential advantage in terms of core quality. However, unspecified bilateral biopsies are commonly submitted in many centers. The length of the core is the key quality indicator of prostate biopsies. Because there are few recent publications comparing the quality of 12 site-designated biopsies versus pooled biopsies, we compared the lengths of the biopsies obtained by both methods.

**Methods:**

The material was obtained from 471 consecutive subjects who underwent prostatic needle biopsy in the Tampere University Hospital district between January and June 2013. Biopsies from 344 subjects fulfilled the inclusion criteria. The total number of cores obtained was 4047. The core lengths were measured on microscope slides. Extraprostatic tissue was subtracted from the core length.

**Results:**

The aggregate lengths observed were 129.5 ± 21.8 mm (mean ± SD) for site-designated cores and 136.9 ± 26.4 mm for pooled cores (p = 0.09). The length of the core was 10.8 ± 1.8 mm for site-designated cores and 11.4 ± 2.2 mm for pooled cores (p = 0.87). The median length for pooled cores was 11 mm (range 5 mm – 18 mm). For individual site-designated cores, the median length was 11 mm (range 7 mm −15 mm). The core length was not correlated with the number of cores embedded into one paraffin block (r = 0.015). There was no significant difference in cancer detection rate (p = 0.62).

**Conclusions:**

Our results suggest that unspecified bilateral biopsies do not automatically lead to reduced core length. We conclude that carefully embedded multiple (three to nine) cores per block may yield cores of equal quality in a more cost-efficient way and that current guidelines favoring individually submitted cores may be too strict.

## Background

The diagnosis of prostatic adenocarcinoma is based on the histopathological findings obtained from prostatic needle biopsies. There is a lot of debate on the best protocol for submitting and labeling of prostate biopsies. According to several current guidelines, individual site-designated biopsies submitted in separate vials are preferred, as they are thought to give better quality samples in terms of tissue fragmentation as well as core length [1,2]. However, it is a common practice to submit unspecified bilateral biopsies both in the U.S. and in Europe [3,4]. The length of the biopsy core is the key quality indicator of a successful biopsy, which influences cancer detection rates and the estimation of prognostic parameters [5-7]. Currently, the recommended procedure is to take five to six biopsies from each side [1,2]. Specifically, additional laterally targeted biopsies have been shown to detect 31% more cancers when compared to the sextant biopsy protocol [5]. The role of augmented biopsy protocols is still controversial. It has been suggested that there is no advantage to taking extended (20 cores) or saturation biopsies (24 cores) in the initial biopsy [8,9]. However, a recent meta-analysis has shown that initial diagnostic saturation biopsies may be warranted for patients with low PSA-values or high-volume prostates [10].

In terms of biopsy quality, it has been suggested that up to three cores could be safely embedded in one paraffin block without compromising the biopsy quality [11]. Currently, approximately half of the pathology laboratories in Europe receive unspecified bilateral biopsies together with individually submitted targeted biopsies from a distinct nodule, while only 40% of laboratories receive all biopsies in separate vials [4]. In the U.S., it is slightly more common to submit site-designated biopsies [3]. Compared to pooled biopsies, a submission of 12 site-designated biopsy cores by the urologist increases the workload for pathology laboratories. The advantage of site-designated biopsies is that localization information is spared, which is important for active surveillance follow-up protocols and helps the urologist to plan surgeries. The quality of needle biopsies is operator dependent, but the main result (i.e., how the tissue looks on a slide) is also dependent upon the pathology laboratory [12]. A recent guideline by the pathology committee of the European Randomized Study of Screening for Prostate Cancer (ERSPC) highlights the importance of special techniques in processing and (pre-)embedding for preserving the quality of the biopsy [13]. Such techniques include the use of sponges to flatten the cores during fixation and dehydration, and the use of metal tampers for the embedding process.

The aim of this study was to determine whether there is a quality difference between site-designated individually embedded and unspecified bilateral (pooled) biopsies, using core length as the main quality indicator and cancer detection rate as a secondary measure. It was hypothesized, that pooling samples in the same biopsy container and resulting paraffin block does not affect the quality of the biopsy. Pooling biopsies reduces the workload of laboratory technicians and pathologists, so if pooled samples are of similar quality than site-designated biopsies, it would be possible to get the same results with less effort.

## Methods

**Figure 1:**
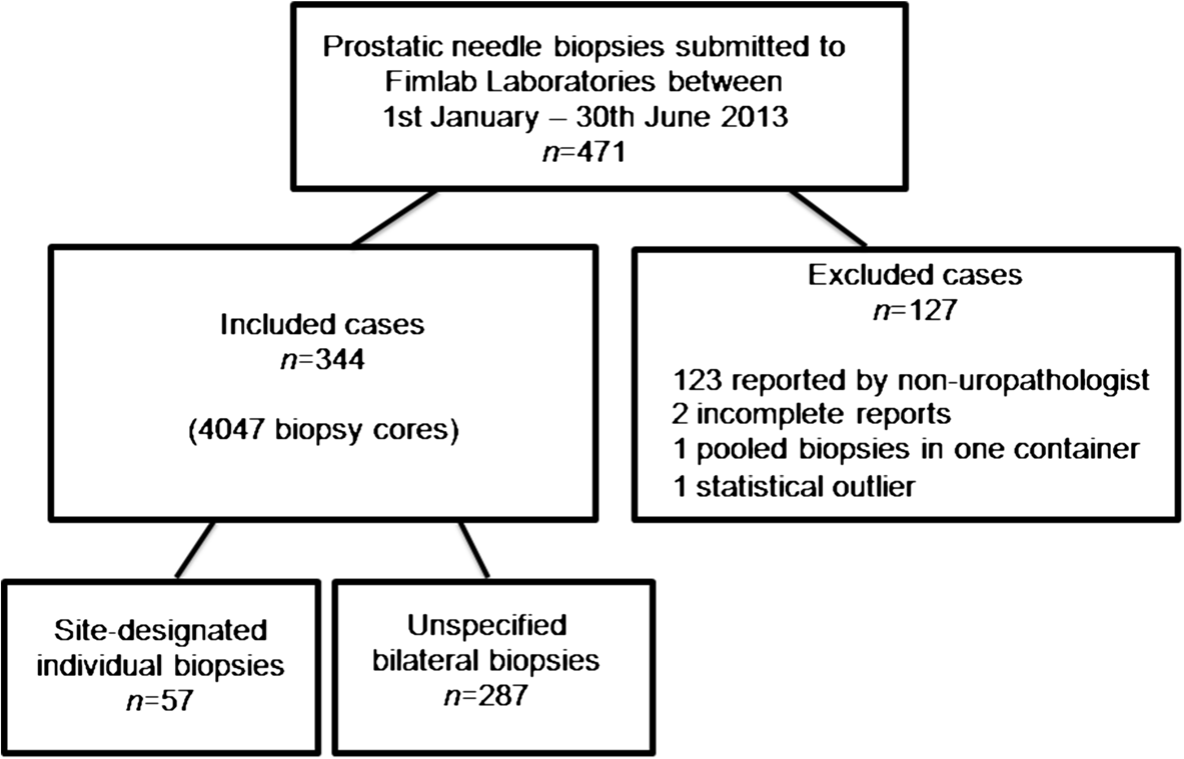
**Study flow chart.** The inclusion criteria were as follows: 1. the biopsy was reported by an uropathologist, 2. bilateral pooled biopsies in two formalin vials or 12 individually submitted cores, 3. the core lengths were measured and reported in millimeters in a standardized manner.

The study was approved by the Ethical Committee of Tampere University Hospital (TAUH), reference number R03203. The material was obtained from 471 consecutive prostate biopsies submitted to Fimlab Laboratories for evaluation during a half year period from January to June 2013. The biopsies were taken in the Tampere University Hospital (TAUH) district by several urologists under standard operating procedure. All the obtained prostate biopsies were evaluated with the following inclusion criteria: 1) the biopsy was reported by one of our five uropathologists (ML, MT, SH, PK, TT), 2) the biopsies were comprised of either 12 individually submitted cores or bilateral pooled biopsies submitted in two formalin vials (plus an extra vial containing one core from a distinct nodule in some cases), and 3) all cores were measured in millimeters and reported in a standardized manner (see later section). Biopsies from 344 subjects fulfilled the inclusion criteria and yielded a total number of 4047 biopsy cores. All of the accepted site-designated biopsies consisted of a set of 12 biopsy containers with a single biopsy inside, except for one case in which only 11 containers were submitted because there were erroneously two biopsies in one vial. A total of 127 cases were excluded. Although it met the inclusion criteria, one case with 12 individually processed cores containing only 20 mm intraprostatic tissue was excluded as a statistical outlier. The inclusion procedure is presented schematically in Figure 1.

All biopsies were taken transrectally with ultrasonography guidance using an 18-gauge needle biopsy gun with an 18-mm sample notch (Bard peripheral vascular, Temple, AZ, U.S.A., ref no. MC 1825) and a side-fire probe. Biopsies were put into vials containing 10% neutral-buffered formalin straight from the biopsy needle. Biopsies from a single patient were transported to our laboratory either in 12 separate vials or in two vials containing several biopsy cores (median number of cores per container was six). The number of submitted vials depended on how the urologist performed the prostate biopsy. All biopsies were processed in Fimlab Laboratories, Tampere University Hospital, in Tampere, Finland.

Site-designated individual biopsies were transferred to separate tissue cassettes in which they were straightened (not stretched) and flattened between sponges during standard dehydration and microwave processing. Pooled biopsies from one vial were treated equally but remained pooled (e.g., multiple straightened cores were sandwiched between sponges into one cassette). Two to four sections were cut from the individual cores and transferred to one slide, depending on the technologist’s visual impression. Because pooled biopsies may have more planar variation inside the paraffin blocks, they were cut on four levels which resulted in the generation of two slides. The blocks were not cut through and step sections were not collected because our current protocol offers residual material for potential immunostaining in most cases.

**Figure 2:**
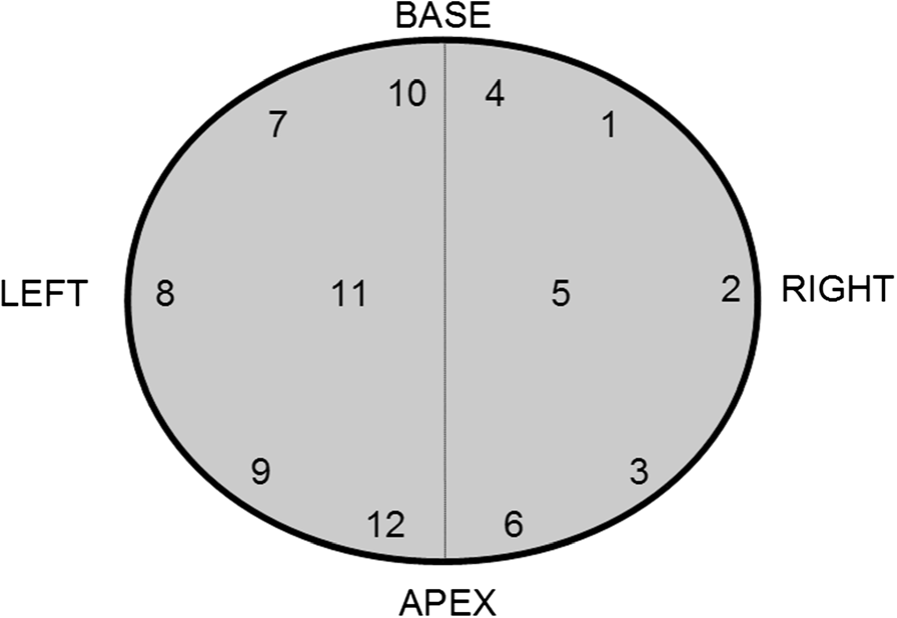
**Biopsy protocol for 12 individually submitted site-designated biopsy cores.**

**Figure 3:**
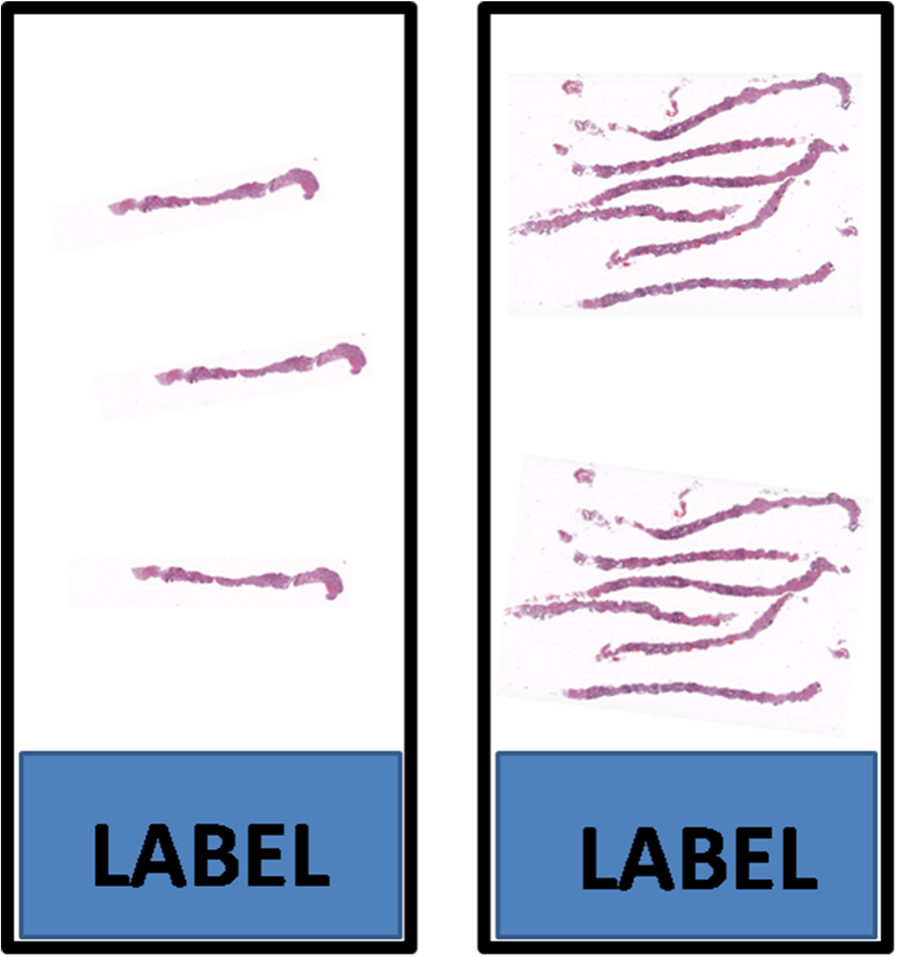
**Two H&E-stained microscopy slides illustrating the visual appearance of individually embedded and pooled biopsies.** On the left: individual biopsy core sectioned at three levels. On the right: six biopsy cores embedded into one paraffin block and sectioned at two levels.

The lengths of the biopsy cores were collected from pathology reports. For individually processed biopsies, the lengths were reported for each biopsy core in millimeters. The 12 loci of individual biopsies were standardized as follows: 1–3 were right lateral base, mid and apex, 4–6 were right medial base, mid and apex, 7–9 were left lateral base, mid and apex, and 10–12 were left medial base, mid and apex, respectively. The current scheme for site-specific needle biopsies in the TAUH district is presented in Figure 2. In the case of multiple biopsies per paraffin block, the information regarding the number of biopsies in the block and the total length of the biopsies in millimeters was required. Separately submitted additional cores targeting a region of palpable resistance were excluded from the length measurements but were included in the cancer detection rate. The length of the core and the possible length of the cancerous tissue were measured from hematoxylin-eosin (H&E) -stained slides either under a light microscope as multiples of 4x/10x objectives visual field diameter or by a liner, depending on the extent of the cancer. According to our standardized protocol, extraprostatic tissue was subtracted from the total core length to obtain the most accurate percentage of cancer. Tissue was considered extraprostatic when containing obvious fat or loose mesenchymal tissue that was distinct from the (pseudo)capsule. For cancerous prostates, a standardized scoring table was applied. The recorded parameters included primary and secondary Gleason patterns, the number of positive cores/total number of cores, cancer length/total length, the percentage of cancer, high grade prostatic intraepithelial neoplasia, and perineural invasion. The microscopic appearance of slides with individually embedded and pooled biopsies are represented in Figure 3.

### Statistical analysis

Data were analyzed using a two-tailed Wilcoxon-Mann–Whitney test to compare aggregate and single biopsy length means and a two-tailed Fisher’s exact test to compare cancer detection rates. The impact of the number of cores embedded in a single paraffin block on the mean length of cores in the corresponding block was tested using Pearson’s correlation coefficient analysis. Statistical significance was considered at p < 0.05.

## Results

Individual site-designated biopsies were submitted to our laboratory in 57 (16.6%) cases, and non-specified (pooled) bilateral biopsies were submitted in 287 (83.4%) cases. Of the pooled biopsies, exactly six plus six cores from the right and left sides were obtained 188 (65.5%) times. More than 12 biopsies were submitted in 38 (13.2%) cases, and less than 10 biopsies were submitted in 18 (6.2%) cases. The minimum number of biopsy cores per subject was 6 (n = 1), and the maximum number was 15 (n = 7).

The mean aggregate length of all biopsy cores from one subject was 133.6 ± 29.9 mm (mean ± SD). No statistically significant difference in the mean aggregate lengths was noted between site-designated and pooled (6 + 6) biopsies or between benign and malignant biopsies (Table 1).

**Table 1:** **Comparison of aggregate lengths of the biopsy cores**

	***n***	**Aggregate length, mm (mean ± SD)**
All	344	133.6 ± 29.9
Site-designated	57	129.5 ± 21.8^1^
Pooled 6 + 6	188	136.9 ± 26.4^1^
Benign	151	132.7 ± 29.3^2^
Malignant	193	134.2 ± 30.4^2^

^1^No statistically significant difference was noted between 12 site-designated cores and unspecified bilateral (6 + 6) cores, p = 0.09.

^2^No statistically significant difference was noted between benign and malignant cases, p = 0.25.

The average core length was 11.4 ± 2.2 mm for pooled biopsies and 10.8 ± 1.8 mm for individually processed cores (p = 0.87). The median length for pooled cores was 11 mm (range 5 mm - 18 mm). For individual site-designated cores the median length was 11 mm (range 7 mm - 15 mm). Because the number of biopsy cores in the vials varied from 1 to 9, we also tested whether increasing the number of cores in a paraffin block would have an adverse effect on the mean length of the cores as one might expect. However, no correlation was noted (Table 2).

**Table 2:** **The impact of the number of biopsy cores embedded into one paraffin block to the mean length of cores in the corresponding block** ^**1**^

**No. of cores in paraffin block**	***n***	**Length of cores, mm (mean ± SD)**
1	684	10.8 ± 1.8
3	8	12.9 ± 2.1
4	20	10.6 ± 2.7
5	75	11.0 ± 2.9
6	417	11.5 ± 2.4
7	40	11.8 ± 2.5
8	10	11.5 ± 2.1
9	2	11.1 ± 1.2

^1^The length of biopsies was not correlated to the number of cores in the block, r = 0.015.

The overall cancer detection rate was 55.8%. Adenocarcinoma was detected in 28/57 (49.1%) cases with site-designated biopsies and in 164/287 (57.1%) cases with pooled biopsies (p = 0.62).

## Discussion

A recent survey regarding the handling of prostate biopsies by the European Network for Uropathologists (ENUP) showed that there is a wide diversity among European pathology centers in the handling of prostate biopsy specimens [4]. The number of biopsy cores taken, the number of cores in a formalin vial, and the (pre-)embedding methods are all variable. Multiple biopsies per vial (and paraffin block) were used in approximately half of the centers that participated in the survey, which is not recommended due to a presumption that tissue quality will be compromised. In our study, there was no difference in the lengths of biopsies regardless of whether they were processed individually or pooled. Furthermore, cancer detection rates were approximately equal between the groups. The slightly lower cancer detection rate noted in individually processed biopsies may be related to the different indications for a biopsy procedure between the groups. However, the observed overall cancer detection rate of 55.8% is quite high. Previously, in a Finnish prostate cancer screening study conducted as a part of the European Randomized Study of Screening for Prostate Cancer (ERSPC), the observed cancer detection rate at a PSA cutoff level of 4 μg/l was found to be 27% [14].

There are several possible reasons for the high cancer detection rate observed in this study. The population included in the study has a relatively high PSA screening frequency due to the prostate cancer screening trial. Some of the patients may have PSA data going back to the start of screening trial in 1996, which has lead to a higher threshold for taking biopsies. Also, the PSA value is no longer considered the only indication for taking a prostate biopsy; more significance is given to the value of free PSA per total PSA. Another reason for the high cancer detection rate may be that some of biopsies are taken from patients in an active follow-up. Finally, there might be skewness in the results due to the inclusion criteria. Benign biopsies are not always reported with the same accuracy as cancer cases, and this inaccuracy in reporting of biopsy length may have disqualified some benign cases from inclusion in this study. Also, urgent cases with a high suspicion of cancer are more likely to be reported by one of the uropathologists conducting the study, which may increase the overall cancer detection percentage.

Our results suggest that the problems encountered with multiple biopsies in one container can be overcome by special tissue pre-embedding methods, including straightening and flattening the cores between sponges before tissue processing and by paying attention to the laboratory technologist’s education. According to the ERSPC pathology committee’s newest guidelines, up to three cores can be safely embedded in a single paraffin block without significant tissue loss [13]. Our results suggest that the maximum number of cores that can safely be embedded in a single paraffin block may be a matter of technique – if one is able to embed single cores well enough, why would it not work for the core next to it? In fact, in one of our earlier experiments, we embedded twelve biopsies in one paraffin block and obtained a satisfactory visual appearance. However, the technique was abandoned because there were two obvious disadvantages: the orientation of the biopsies in the paraffin block needed to be diagonal instead of longitudinal which made sectioning more difficult, and the tips of the cores stayed outside of the staining area of the automated immunostaining system due to their marginal position.

According to Bostwick *et al*., the mean length of prostate biopsies in Western Europe was 13.1 mm at the entry level of their study [12]. In the present study, the mean length of the core was shorter (11.4 mm). However, the aforementioned values are not comparable because in our study extraprostatic tissue was subtracted to obtain the most accurate percentage of cancer tissue possible.

**Figure 4:**
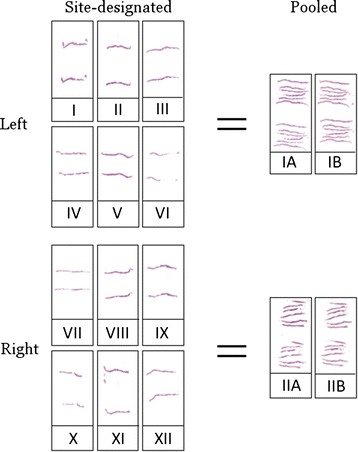
**Two complete slide sets illustrating the different workload of site-designated and pooled biopsies.** Individual biopsy cores yield twelve slides per subject whereas pooled biopsies yield only four slides.

It is likely that the most important advantage of individually embedded biopsies is not the biopsy core quality but rather the spared locus information. This is an important issue in selected cases, and site-designated biopsies should be encouraged. On the other hand, the use of multiple biopsies per vial (and paraffin block) is supported by less extensive laboratory loading and better facilities for immunohistochemistry. Our medium-sized laboratory receives biopsies from approximately 1000 patients per year. Widespread use of site-designated biopsies would annually increase the number of paraffin blocks by approximately 10,000, which increases the workload for the pathology laboratory throughout various steps including processing, embedding, sectioning and analyzing. Roughly estimated this would take approximately 80 working days for sectioning only and would increase the time pathologists spend analyzing and reporting prostate biopsies (Figure 4).

There are several limitations in the present study. First, the prostate biopsies, although taken with same equipment, may have variations due to different urologists who performed the biopsies. Second, the slides were not re-evaluated. Additionally, by digitizing all of the material and measuring core areas instead of the lengths of the biopsies, the quality indicator would have been more accurate. In the present study we preferred to use our current methods because at the moment there are no area-based prognostic nomograms available, and measuring the actual tissue from histological slides is the gold standard. Third, the subtraction of extraprostatic tissue is somewhat subjective. However, this subjectivity should be equally transferred to both types of specimens. Finally, the amount of site-designated individual biopsies only represented 17% of the studied cases and the imbalance between the groups may cause unreliability in the final results. As a limitation of this study, it must be pointed out, that this study only shows the situation in our facility and to determine the real impact in a clinical context, a multicenter study would describe the overall situation of prostate biopsies better. Our aim was simply to determine whether there were fundamental differences in favor of either method.

## Conclusions

The number of submitted vials depends on how the urologist performs the prostate biopsy. We have received both individually submitted and pooled biopsies for several years without noticing an obvious difference in their quality. However, the workload for laboratory technologists and pathologists is substantially higher for site-designated individually embedded biopsy cores. In our material, we did not find evidence regarding the superior quality of the individually submitted and embedded biopsies. We conclude that the current recommendations favoring site-designated biopsies may be too strict - the guidelines should probably recommend a result rather than a process.
